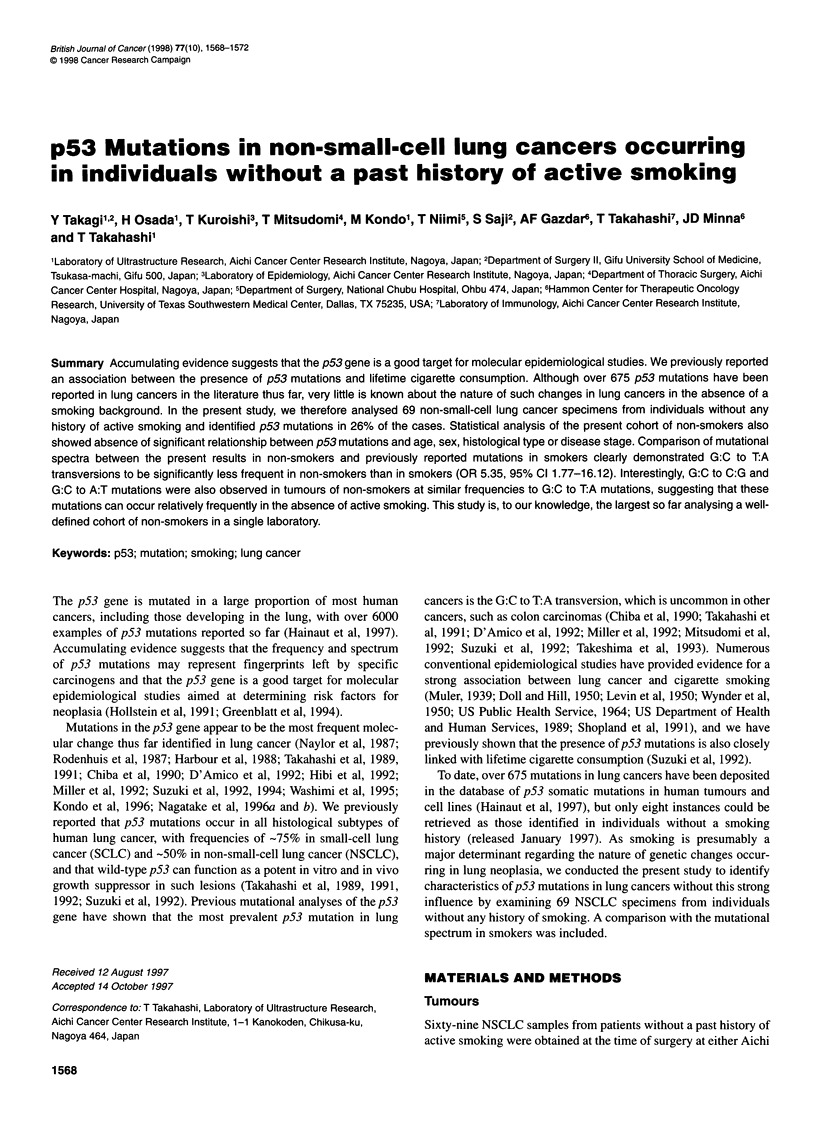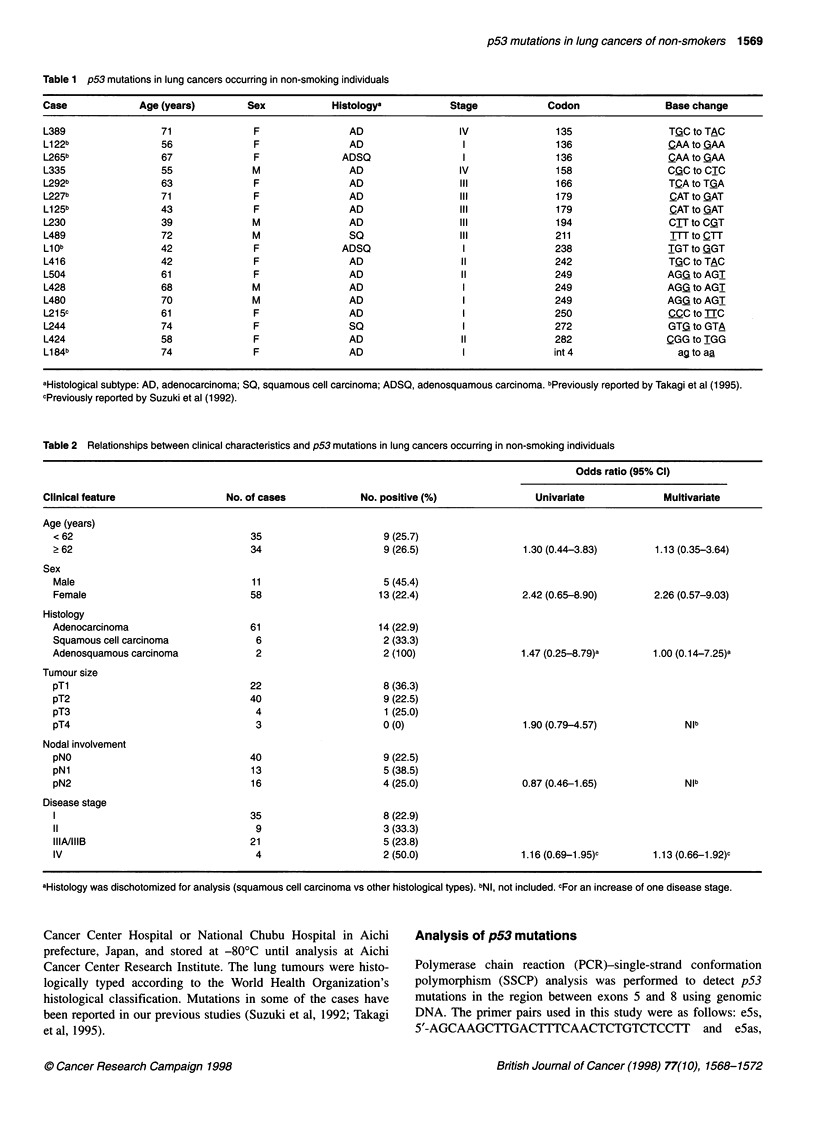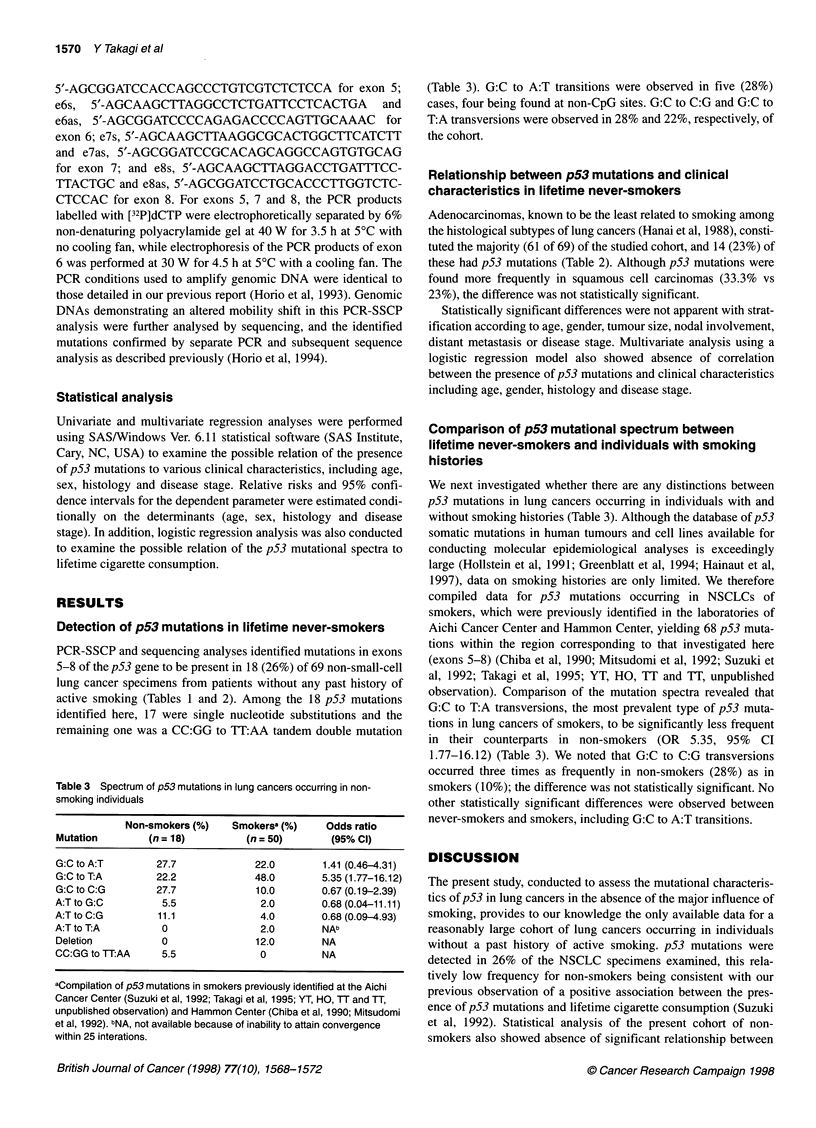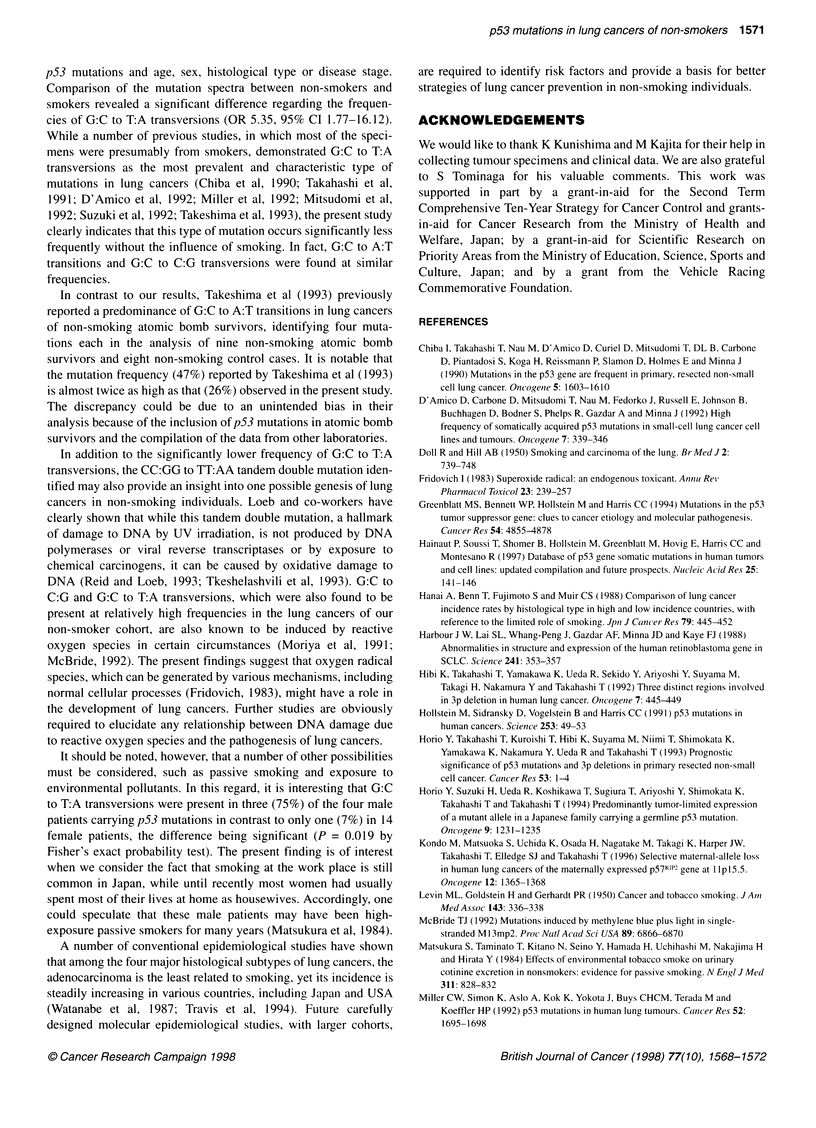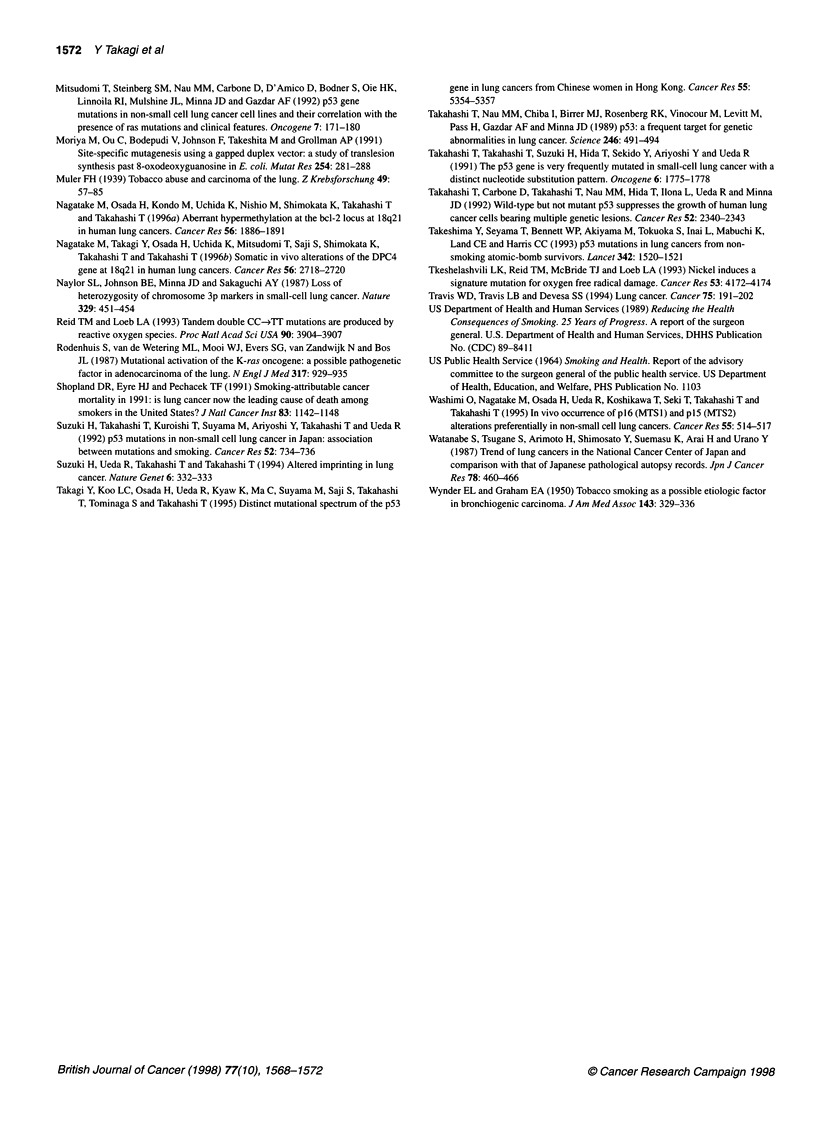# p53 mutations in non-small-cell lung cancers occurring in individuals without a past history of active smoking.

**DOI:** 10.1038/bjc.1998.258

**Published:** 1998-05

**Authors:** Y. Takagi, H. Osada, T. Kuroishi, T. Mitsudomi, M. Kondo, T. Niimi, S. Saji, A. F. Gazdar, T. Takahashi, J. D. Minna, T. Takahashi

**Affiliations:** Laboratory of Ultrastructure Research, Aichi Cancer Center Research Institute, Nagoya, Japan.

## Abstract

Accumulating evidence suggests that the p53 gene is a good target for molecular epidemiological studies. We previously reported an association between the presence of p53 mutations and lifetime cigarette consumption. Although over 675 p53 mutations have been reported in lung cancers in the literature thus far, very little is known about the nature of such changes in lung cancers in the absence of a smoking background. In the present study, we therefore analysed 69 non-small-cell lung cancer specimens from individuals without any history of active smoking and identified p53 mutations in 26% of the cases. Statistical analysis of the present cohort of non-smokers also showed absence of significant relationship between p53 mutations and age, sex, histological type or disease stage. Comparison of mutational spectra between the present results in non-smokers and previously reported mutations in smokers clearly demonstrated G:C to T:A transversions to be significantly less frequent in non-smokers than in smokers (OR 5.35, 95% CI 1.77-16.12). Interestingly, G:C to C:G and G:C to A:T mutations were also observed in tumours of non-smokers at similar frequencies to G:C to T:A mutations, suggesting that these mutations can occur relatively frequently in the absence of active smoking. This study is, to our knowledge, the largest so far analysing a well-defined cohort of non-smokers in a single laboratory.


British Journal of Cancer (1998) 77(10), 1568–1572
© 1998 Cancer Research Campaign

### p53 Mutations in non-small-cell lung cancers occurring in individuals without a past history of active smoking

**Y Takagi[1,2], H Osada[1], T Kuroishi[3], T Mitsudomi[4], M Kondo[1], T Niimi[5], S Saji[2], AF Gazdar[6], T Takahashi[7], JD Minna[6] and T Takahashi[1]**

[1]Laboratory of Ultrastructure Research, Aichi Cancer Center Research Institute, Nagoya, Japan; [2]Department of Surgery II, Gifu University School of Medicine, Tsukasa-machi, Gifu 500, Japan; [3]Laboratory of Epidemiology, Aichi Cancer Center Research Institute, Nagoya, Japan; [4]Department of Thoracic Surgery, Aichi Cancer Center Hospital, Nagoya, Japan; [5]Department of Surgery, National Chubu Hospital, Ohbu 474, Japan; [6]Hammon Center for Therapeutic Oncology Research, University of Texas Southwestern Medical Center, Dallas, TX 75235, USA; [7]Laboratory of Immunology, Aichi Cancer Center Research Institute, Nagoya, Japan

**Summary** Accumulating evidence suggests that the *p53* gene is a good target for molecular epidemiological studies. We previously reported an association between the presence of *p53* mutations and lifetime cigarette consumption. Although over 675 *p53* mutations have been reported in lung cancers in the literature thus far, very little is known about the nature of such changes in lung cancers in the absence of a smoking background. In the present study, we therefore analysed 69 non-small-cell lung cancer specimens from individuals without any history of active smoking and identified *p53* mutations in 26% of the cases. Statistical analysis of the present cohort of non-smokers also showed absence of significant relationship between *p53* mutations and age, sex, histological type or disease stage. Comparison of mutational spectra between the present results in non-smokers and previously reported mutations in smokers clearly demonstrated G:C to T:A transversions to be significantly less frequent in non-smokers than in smokers (OR 5.35, 95% CI 1.77–16.12). Interestingly, G:C to C:G and G:C to A:T mutations were also observed in tumours of non-smokers at similar frequencies to G:C to T:A mutations, suggesting that these mutations can occur relatively frequently in the absence of active smoking. This study is, to our knowledge, the largest so far analysing a well-defined cohort of non-smokers in a single laboratory.

**Keywords:** p53; mutation; smoking; lung cancer

The *p53* gene is mutated in a large proportion of most human cancers, including those developing in the lung, with over 6000 examples of *p53* mutations reported so far (Hainaut et al, 1997). Accumulating evidence suggests that the frequency and spectrum of *p53* mutations may represent fingerprints left by specific carcinogens and that the *p53* gene is a good target for molecular epidemiological studies aimed at determining risk factors for neoplasia (Hollstein et al, 1991; Greenblatt et al, 1994).

Mutations in the *p53* gene appear to be the most frequent molecular change thus far identified in lung cancer (Naylor et al, 1987; Rodenhuis et al, 1987; Harbour et al, 1988; Takahashi et al, 1989, 1991; Chiba et al, 1990; D'Amico et al, 1992; Hibi et al, 1992; Miller et al, 1992; Suzuki et al, 1992, 1994; Washimi et al, 1995; Kondo et al, 1996; Nagatake et al, 1996*a* and *b*). We previously reported that *p53* mutations occur in all histological subtypes of human lung cancer, with frequencies of ~75% in small-cell lung cancer (SCLC) and ~50% in non-small-cell lung cancer (NSCLC), and that wild-type *p53* can function as a potent in vitro and in vivo growth suppressor in such lesions (Takahashi et al, 1989, 1991, 1992; Suzuki et al, 1992). Previous mutational analyses of the *p53* gene have shown that the most prevalent *p53* mutation in lung cancers is the G:C to T:A transversion, which is uncommon in other cancers, such as colon carcinomas (Chiba et al, 1990; Takahashi et al, 1991; D'Amico et al, 1992; Miller et al, 1992; Mitsudomi et al, 1992; Suzuki et al, 1992; Takeshima et al, 1993). Numerous conventional epidemiological studies have provided evidence for a strong association between lung cancer and cigarette smoking (Muler, 1939; Doll and Hill, 1950; Levin et al, 1950; Wynder et al, 1950; US Public Health Service, 1964; US Department of Health and Human Services, 1989; Shopland et al, 1991), and we have previously shown that the presence of *p53* mutations is also closely linked with lifetime cigarette consumption (Suzuki et al, 1992).

To date, over 675 mutations in lung cancers have been deposited in the database of *p53* somatic mutations in human tumours and cell lines (Hainaut et al, 1997), but only eight instances could be retrieved as those identified in individuals without a smoking history (released January 1997). As smoking is presumably a major determinant regarding the nature of genetic changes occurring in lung neoplasia, we conducted the present study to identify characteristics of *p53* mutations in lung cancers without this strong influence by examining 69 NSCLC specimens from individuals without any history of smoking. A comparison with the mutational spectrum in smokers was included.

### MATERIALS AND METHODS

### Tumours

Sixty-nine NSCLC samples from patients without a past history of active smoking were obtained at the time of surgery at either Aichi

*Received 12 August 1997*
*Accepted 14 October 1997*

*Correspondence to:* T Takahashi, Laboratory of Ultrastructure Research, Aichi Cancer Center Research Institute, 1–1 Kanokoden, Chikusa-ku, Nagoya 464, Japan

**Table 1** *p53* mutations in lung cancers occurring in non-smoking individuals

| Case | Age (years) | Sex | Histology[a] | Stage | Codon | Base change |
|---|---|---|---|---|---|---|
| L389 | 71 | F | AD | IV | 135 | TGC to TAC |
| L122[b] | 56 | F | AD | I | 136 | CAA to GAA |
| L265[b] | 67 | F | ADSQ | I | 136 | CAA to GAA |
| L335 | 55 | M | AD | IV | 158 | CGC to CTC |
| L292[b] | 63 | F | AD | III | 166 | TCA to TGA |
| L227[b] | 71 | F | AD | III | 179 | CAT to GAT |
| L125[b] | 43 | F | AD | III | 179 | CAT to GAT |
| L230 | 39 | M | AD | III | 194 | CTT to CGT |
| L489 | 72 | M | SQ | III | 211 | TTT to CTT |
| L10[b] | 42 | F | ADSQ | I | 238 | TGT to GGT |
| L416 | 42 | F | AD | II | 242 | TGC to TAC |
| L504 | 61 | F | AD | II | 249 | AGG to AGT |
| L428 | 68 | M | AD | I | 249 | AGG to AGT |
| L480 | 70 | M | AD | I | 249 | AGG to AGT |
| L215[c] | 61 | F | AD | I | 250 | CCC to TTC |
| L244 | 74 | F | SQ | I | 272 | GTG to GTA |
| L424 | 58 | F | AD | II | 282 | CGG to TGG |
| L184[b] | 74 | F | AD | I | int 4 | ag to aa |

[a]Histological subtype: AD, adenocarcinoma; SQ, squamous cell carcinoma; ADSQ, adenosquamous carcinoma. [b]Previously reported by Takagi et al (1995). [c]Previously reported by Suzuki et al (1992).

**Table 2** Relationships between clinical characteristics and *p53* mutations in lung cancers occurring in non-smoking individuals

| | | | Odds ratio (95% CI) | |
|---|---|---|---|---|
| Clinical feature | No. of cases | No. positive (%) | Univariate | Multivariate |
| Age (years) | | | | |
| < 62 | 35 | 9 (25.7) | | |
| ≥ 62 | 34 | 9 (26.5) | 1.30 (0.44–3.83) | 1.13 (0.35–3.64) |
| Sex | | | | |
| Male | 11 | 5 (45.4) | | |
| Female | 58 | 13 (22.4) | 2.42 (0.65–8.90) | 2.26 (0.57–9.03) |
| Histology | | | | |
| Adenocarcinoma | 61 | 14 (22.9) | | |
| Squamous cell carcinoma | 6 | 2 (33.3) | | |
| Adenosquamous carcinoma | 2 | 2 (100) | 1.47 (0.25–8.79)[a] | 1.00 (0.14–7.25)[a] |
| Tumour size | | | | |
| pT1 | 22 | 8 (36.3) | | |
| pT2 | 40 | 9 (22.5) | | |
| pT3 | 4 | 1 (25.0) | | |
| pT4 | 3 | 0 (0) | 1.90 (0.79–4.57) | NI[b] |
| Nodal involvement | | | | |
| pN0 | 40 | 9 (22.5) | | |
| pN1 | 13 | 5 (38.5) | | |
| pN2 | 16 | 4 (25.0) | 0.87 (0.46–1.65) | NI[b] |
| Disease stage | | | | |
| I | 35 | 8 (22.9) | | |
| II | 9 | 3 (33.3) | | |
| IIIA/IIIB | 21 | 5 (23.8) | | |
| IV | 4 | 2 (50.0) | 1.16 (0.69–1.95)[c] | 1.13 (0.66–1.92)[c] |

[a]Histology was dischotomized for analysis (squamous cell carcinoma vs other histological types). [b]NI, not included. [c]For an increase of one disease stage.

Cancer Center Hospital or National Chubu Hospital in Aichi prefecture, Japan, and stored at –80°C until analysis at Aichi Cancer Center Research Institute. The lung tumours were histologically typed according to the World Health Organization's histological classification. Mutations in some of the cases have been reported in our previous studies (Suzuki et al, 1992; Takagi et al, 1995).

### Analysis of *p53* mutations

Polymerase chain reaction (PCR)–single-strand conformation polymorphism (SSCP) analysis was performed to detect *p53* mutations in the region between exons 5 and 8 using genomic DNA. The primer pairs used in this study were as follows: e5s, 5′-AGCAAGCTTGACTTTCAACTCTGTCTCCTT and e5as,

© Cancer Research Campaign 1998

5′-AGCGGATCCACCAGCCCTGTCGTCTCTCCA for exon 5; e6s, 5′-AGCAAGCTTAGGCCTCTGATTCCTCACTGA and e6as, 5′-AGCGGATCCCCAGAGACCCCAGTTGCAAAC for exon 6; e7s, 5′-AGCAAGCTTAAGGCGCACTGGCTTCATCTT and e7as, 5′-AGCGGATCCGCACAGCAGGCCAGTGTGCAG for exon 7; and e8s, 5′-AGCAAGCTTAGGACCTGATTTCC-TTACTGC and e8as, 5′-AGCGGATCCTGCACCCTTGGTCTC-CTCCAC for exon 8. For exons 5, 7 and 8, the PCR products labelled with [$^{32}$P]dCTP were electrophoretically separated by 6% non-denaturing polyacrylamide gel at 40 W for 3.5 h at 5°C with no cooling fan, while electrophoresis of the PCR products of exon 6 was performed at 30 W for 4.5 h at 5°C with a cooling fan. The PCR conditions used to amplify genomic DNA were identical to those detailed in our previous report (Horio et al, 1993). Genomic DNAs demonstrating an altered mobility shift in this PCR-SSCP analysis were further analysed by sequencing, and the identified mutations confirmed by separate PCR and subsequent sequence analysis as described previously (Horio et al, 1994).

### Statistical analysis

Univariate and multivariate regression analyses were performed using SAS/Windows Ver. 6.11 statistical software (SAS Institute, Cary, NC, USA) to examine the possible relation of the presence of *p53* mutations to various clinical characteristics, including age, sex, histology and disease stage. Relative risks and 95% confidence intervals for the dependent parameter were estimated conditionally on the determinants (age, sex, histology and disease stage). In addition, logistic regression analysis was also conducted to examine the possible relation of the *p53* mutational spectra to lifetime cigarette consumption.

### RESULTS

### Detection of *p53* mutations in lifetime never-smokers

PCR-SSCP and sequencing analyses identified mutations in exons 5–8 of the *p53* gene to be present in 18 (26%) of 69 non-small-cell lung cancer specimens from patients without any past history of active smoking (Tables 1 and 2). Among the 18 *p53* mutations identified here, 17 were single nucleotide substitutions and the remaining one was a CC:GG to TT:AA tandem double mutation (Table 3). G:C to A:T transitions were observed in five (28%) cases, four being found at non-CpG sites. G:C to C:G and G:C to T:A transversions were observed in 28% and 22%, respectively, of the cohort.

**Table 3** Spectrum of *p53* mutations in lung cancers occurring in non-smoking individuals

| Mutation | Non-smokers (%) (*n* = 18) | Smokers[a] (%) (*n* = 50) | Odds ratio (95% CI) |
|---|---|---|---|
| G:C to A:T | 27.7 | 22.0 | 1.41 (0.46–4.31) |
| G:C to T:A | 22.2 | 48.0 | 5.35 (1.77–16.12) |
| G:C to C:G | 27.7 | 10.0 | 0.67 (0.19–2.39) |
| A:T to G:C | 5.5 | 2.0 | 0.68 (0.04–11.11) |
| A:T to C:G | 11.1 | 4.0 | 0.68 (0.09–4.93) |
| A:T to T:A | 0 | 2.0 | NA[b] |
| Deletion | 0 | 12.0 | NA |
| CC:GG to TT:AA | 5.5 | 0 | NA |

[a]Compilation of *p53* mutations in smokers previously identified at the Aichi Cancer Center (Suzuki et al, 1992; Takagi et al, 1995; YT, HO, TT and TT, unpublished observation) and Hammon Center (Chiba et al, 1990; Mitsudomi et al, 1992). [b]NA, not available because of inability to attain convergence within 25 interations.

### Relationship between *p53* mutations and clinical characteristics in lifetime never-smokers

Adenocarcinomas, known to be the least related to smoking among the histological subtypes of lung cancers (Hanai et al, 1988), constituted the majority (61 of 69) of the studied cohort, and 14 (23%) of these had *p53* mutations (Table 2). Although *p53* mutations were found more frequently in squamous cell carcinomas (33.3% vs 23%), the difference was not statistically significant.

Statistically significant differences were not apparent with stratification according to age, gender, tumour size, nodal involvement, distant metastasis or disease stage. Multivariate analysis using a logistic regression model also showed absence of correlation between the presence of *p53* mutations and clinical characteristics including age, gender, histology and disease stage.

### Comparison of *p53* mutational spectrum between lifetime never-smokers and individuals with smoking histories

We next investigated whether there are any distinctions between *p53* mutations in lung cancers occurring in individuals with and without smoking histories (Table 3). Although the database of *p53* somatic mutations in human tumours and cell lines available for conducting molecular epidemiological analyses is exceedingly large (Hollstein et al, 1991; Greenblatt et al, 1994; Hainaut et al, 1997), data on smoking histories are only limited. We therefore compiled data for *p53* mutations occurring in NSCLCs of smokers, which were previously identified in the laboratories of Aichi Cancer Center and Hammon Center, yielding 68 *p53* mutations within the region corresponding to that investigated here (exons 5–8) (Chiba et al, 1990; Mitsudomi et al, 1992; Suzuki et al, 1992; Takagi et al, 1995; YT, HO, TT and TT, unpublished observation). Comparison of the mutation spectra revealed that G:C to T:A transversions, the most prevalent type of *p53* mutations in lung cancers of smokers, to be significantly less frequent in their counterparts in non-smokers (OR 5.35, 95% CI 1.77–16.12) (Table 3). We noted that G:C to C:G transversions occurred three times as frequently in non-smokers (28%) as in smokers (10%); the difference was not statistically significant. No other statistically significant differences were observed between never-smokers and smokers, including G:C to A:T transitions.

### DISCUSSION

The present study, conducted to assess the mutational characteristics of *p53* in lung cancers in the absence of the major influence of smoking, provides to our knowledge the only available data for a reasonably large cohort of lung cancers occurring in individuals without a past history of active smoking. *p53* mutations were detected in 26% of the NSCLC specimens examined, this relatively low frequency for non-smokers being consistent with our previous observation of a positive association between the presence of *p53* mutations and lifetime cigarette consumption (Suzuki et al, 1992). Statistical analysis of the present cohort of non-smokers also showed absence of significant relationship between

© Cancer Research Campaign 1998

*p53* mutations and age, sex, histological type or disease stage. Comparison of the mutation spectra between non-smokers and smokers revealed a significant difference regarding the frequencies of G:C to T:A transversions (OR 5.35, 95% CI 1.77–16.12). While a number of previous studies, in which most of the specimens were presumably from smokers, demonstrated G:C to T:A transversions as the most prevalent and characteristic type of mutations in lung cancers (Chiba et al, 1990; Takahashi et al, 1991; D'Amico et al, 1992; Miller et al, 1992; Mitsudomi et al, 1992; Suzuki et al, 1992; Takeshima et al, 1993), the present study clearly indicates that this type of mutation occurs significantly less frequently without the influence of smoking. In fact, G:C to A:T transitions and G:C to C:G transversions were found at similar frequencies.

In contrast to our results, Takeshima et al (1993) previously reported a predominance of G:C to A:T transitions in lung cancers of non-smoking atomic bomb survivors, identifying four mutations each in the analysis of nine non-smoking atomic bomb survivors and eight non-smoking control cases. It is notable that the mutation frequency (47%) reported by Takeshima et al (1993) is almost twice as high as that (26%) observed in the present study. The discrepancy could be due to an unintended bias in their analysis because of the inclusion of *p53* mutations in atomic bomb survivors and the compilation of the data from other laboratories.

In addition to the significantly lower frequency of G:C to T:A transversions, the CC:GG to TT:AA tandem double mutation identified may also provide an insight into one possible genesis of lung cancers in non-smoking individuals. Loeb and co-workers have clearly shown that while this tandem double mutation, a hallmark of damage to DNA by UV irradiation, is not produced by DNA polymerases or viral reverse transcriptases or by exposure to chemical carcinogens, it can be caused by oxidative damage to DNA (Reid and Loeb, 1993; Tkeshelashvili et al, 1993). G:C to C:G and G:C to T:A transversions, which were also found to be present at relatively high frequencies in the lung cancers of our non-smoker cohort, are also known to be induced by reactive oxygen species in certain circumstances (Moriya et al, 1991; McBride, 1992). The present findings suggest that oxygen radical species, which can be generated by various mechanisms, including normal cellular processes (Fridovich, 1983), might have a role in the development of lung cancers. Further studies are obviously required to elucidate any relationship between DNA damage due to reactive oxygen species and the pathogenesis of lung cancers.

It should be noted, however, that a number of other possibilities must be considered, such as passive smoking and exposure to environmental pollutants. In this regard, it is interesting that G:C to T:A transversions were present in three (75%) of the four male patients carrying *p53* mutations in contrast to only one (7%) in 14 female patients, the difference being significant ($P$ = 0.019 by Fisher's exact probability test). The present finding is of interest when we consider the fact that smoking at the work place is still common in Japan, while until recently most women had usually spent most of their lives at home as housewives. Accordingly, one could speculate that these male patients may have been high-exposure passive smokers for many years (Matsukura et al, 1984).

A number of conventional epidemiological studies have shown that among the four major histological subtypes of lung cancers, the adenocarcinoma is the least related to smoking, yet its incidence is steadily increasing in various countries, including Japan and USA (Watanabe et al, 1987; Travis et al, 1994). Future carefully designed molecular epidemiological studies, with larger cohorts, are required to identify risk factors and provide a basis for better strategies of lung cancer prevention in non-smoking individuals.

### ACKNOWLEDGEMENTS

We would like to thank K Kunishima and M Kajita for their help in collecting tumour specimens and clinical data. We are also grateful to S Tominaga for his valuable comments. This work was supported in part by a grant-in-aid for the Second Term Comprehensive Ten-Year Strategy for Cancer Control and grants-in-aid for Cancer Research from the Ministry of Health and Welfare, Japan; by a grant-in-aid for Scientific Research on Priority Areas from the Ministry of Education, Science, Sports and Culture, Japan; and by a grant from the Vehicle Racing Commemorative Foundation.

### REFERENCES

Chiba I, Takahashi T, Nau M, D'Amico D, Curiel D, Mitsudomi T, DL B, Carbone D, Piantadosi S, Koga H, Reissmann P, Slamon D, Holmes E and Minna J (1990) Mutations in the p53 gene are frequent in primary, resected non-small cell lung cancer. *Oncogene* **5**: 1603–1610

D'Amico D, Carbone D, Mitsudomi T, Nau M, Fedorko J, Russell E, Johnson B, Buchhagen D, Bodner S, Phelps R, Gazdar A and Minna J (1992) High frequency of somatically acquired p53 mutations in small-cell lung cancer cell lines and tumours. *Oncogene* **7**: 339–346

Doll R and Hill AB (1950) Smoking and carcinoma of the lung. *Br Med J* **2**: 739–748

Fridovich I (1983) Superoxide radical: an endogenous toxicant. *Annu Rev Pharmacol Toxicol* **23**: 239–257

Greenblatt MS, Bennett WP, Hollstein M and Harris CC (1994) Mutations in the p53 tumor suppressor gene: clues to cancer etiology and molecular pathogenesis. *Cancer Res* **54**: 4855–4878

Hainaut P, Soussi T, Shomer B, Hollstein M, Greenblatt M, Hovig E, Harris CC and Montesano R (1997) Database of p53 gene somatic mutations in human tumors and cell lines: updated compilation and future prospects. *Nucleic Acid Res* **25**: 141–146

Hanai A, Benn T, Fujimoto S and Muir CS (1988) Comparison of lung cancer incidence rates by histological type in high and low incidence countries, with reference to the limited role of smoking. *Jpn J Cancer Res* **79**: 445–452

Harbour J W, Lai SL, Whang-Peng J, Gazdar AF, Minna JD and Kaye FJ (1988) Abnormalities in structure and expression of the human retinoblastoma gene in SCLC. *Science* **241**: 353–357

Hibi K, Takahashi T, Yamakawa K, Ueda R, Sekido Y, Ariyoshi Y, Suyama M, Takagi H, Nakamura Y and Takahashi T (1992) Three distinct regions involved in 3p deletion in human lung cancer. *Oncogene* **7**: 445–449

Hollstein M, Sidransky D, Vogelstein B and Harris CC (1991) p53 mutations in human cancers. *Science* **253**: 49–53

Horio Y, Takahashi T, Kuroishi T, Hibi K, Suyama M, Niimi T, Shimokata K, Yamakawa K, Nakamura Y, Ueda R and Takahashi T (1993) Prognostic significance of p53 mutations and 3p deletions in primary resected non-small cell cancer. *Cancer Res* **53**: 1–4

Horio Y, Suzuki H, Ueda R, Koshikawa T, Sugiura T, Ariyoshi Y, Shimokata K, Takahashi T and Takahashi T (1994) Predominantly tumor-limited expression of a mutant allele in a Japanese family carrying a germline p53 mutation. *Oncogene* **9**: 1231–1235

Kondo M, Matsuoka S, Uchida K, Osada H, Nagatake M, Takagi K, Harper JW, Takahashi T, Elledge SJ and Takahashi T (1996) Selective maternal-allele loss in human lung cancers of the maternally expressed $p57^{KIP2}$ gene at 11p15.5. *Oncogene* **12**: 1365–1368

Levin ML, Goldstein H and Gerhardt PR (1950) Cancer and tobacco smoking. *J Am Med Assoc* **143**: 336–338

McBride TJ (1992) Mutations induced by methylene blue plus light in single-stranded M13mp2. *Proc Natl Acad Sci USA* **89**: 6866–6870

Matsukura S, Taminato T, Kitano N, Seino Y, Hamada H, Uchihashi M, Nakajima H and Hirata Y (1984) Effects of environmental tobacco smoke on urinary cotinine excretion in nonsmokers: evidence for passive smoking. *N Engl J Med* **311**: 828–832

Miller CW, Simon K, Aslo A, Kok K, Yokota J, Buys CHCM, Terada M and Koeffler HP (1992) p53 mutations in human lung tumours. *Cancer Res* **52**: 1695–1698

© Cancer Research Campaign 1998

Mitsudomi T, Steinberg SM, Nau MM, Carbone D, D'Amico D, Bodner S, Oie HK, Linnoila RI, Mulshine JL, Minna JD and Gazdar AF (1992) p53 gene mutations in non-small cell lung cancer cell lines and their correlation with the presence of ras mutations and clinical features. *Oncogene* **7**: 171–180

Moriya M, Ou C, Bodepudi V, Johnson F, Takeshita M and Grollman AP (1991) Site-specific mutagenesis using a gapped duplex vector: a study of translesion synthesis past 8-oxodeoxyguanosine in *E. coli*. *Mutat Res* **254**: 281–288

Muler FH (1939) Tobacco abuse and carcinoma of the lung. *Z Krebsforschung* **49**: 57–85

Nagatake M, Osada H, Kondo M, Uchida K, Nishio M, Shimokata K, Takahashi T and Takahashi T (1996*a*) Aberrant hypermethylation at the bcl-2 locus at 18q21 in human lung cancers. *Cancer Res* **56**: 1886–1891

Nagatake M, Takagi Y, Osada H, Uchida K, Mitsudomi T, Saji S, Shimokata K, Takahashi T and Takahashi T (1996*b*) Somatic in vivo alterations of the DPC4 gene at 18q21 in human lung cancers. *Cancer Res* **56**: 2718–2720

Naylor SL, Johnson BE, Minna JD and Sakaguchi AY (1987) Loss of heterozygosity of chromosome 3p markers in small-cell lung cancer. *Nature* **329**: 451–454

Reid TM and Loeb LA (1993) Tandem double CC→TT mutations are produced by reactive oxygen species. *Proc Natl Acad Sci USA* **90**: 3904–3907

Rodenhuis S, van de Wetering ML, Mooi WJ, Evers SG, van Zandwijk N and Bos JL (1987) Mutational activation of the K-*ras* oncogene: a possible pathogenetic factor in adenocarcinoma of the lung. *N Engl J Med* **317**: 929–935

Shopland DR, Eyre HJ and Pechacek TF (1991) Smoking-attributable cancer mortality in 1991: is lung cancer now the leading cause of death among smokers in the United States? *J Natl Cancer Inst* **83**: 1142–1148

Suzuki H, Takahashi T, Kuroishi T, Suyama M, Ariyoshi Y, Takahashi T and Ueda R (1992) p53 mutations in non-small cell lung cancer in Japan: association between mutations and smoking. *Cancer Res* **52**: 734–736

Suzuki H, Ueda R, Takahashi T and Takahashi T (1994) Altered imprinting in lung cancer. *Nature Genet* **6**: 332–333

Takagi Y, Koo LC, Osada H, Ueda R, Kyaw K, Ma C, Suyama M, Saji S, Takahashi T, Tominaga S and Takahashi T (1995) Distinct mutational spectrum of the p53 gene in lung cancers from Chinese women in Hong Kong. *Cancer Res* **55**: 5354–5357

Takahashi T, Nau MM, Chiba I, Birrer MJ, Rosenberg RK, Vinocour M, Levitt M, Pass H, Gazdar AF and Minna JD (1989) p53: a frequent target for genetic abnormalities in lung cancer. *Science* **246**: 491–494

Takahashi T, Takahashi T, Suzuki H, Hida T, Sekido Y, Ariyoshi Y and Ueda R (1991) The p53 gene is very frequently mutated in small-cell lung cancer with a distinct nucleotide substitution pattern. *Oncogene* **6**: 1775–1778

Takahashi T, Carbone D, Takahashi T, Nau MM, Hida T, Ilona L, Ueda R and Minna JD (1992) Wild-type but not mutant p53 suppresses the growth of human lung cancer cells bearing multiple genetic lesions. *Cancer Res* **52**: 2340–2343

Takeshima Y, Seyama T, Bennett WP, Akiyama M, Tokuoka S, Inai L, Mabuchi K, Land CE and Harris CC (1993) p53 mutations in lung cancers from non-smoking atomic-bomb survivors. *Lancet* **342**: 1520–1521

Tkeshelashvili LK, Reid TM, McBride TJ and Loeb LA (1993) Nickel induces a signature mutation for oxygen free radical damage. *Cancer Res* **53**: 4172–4174

Travis WD, Travis LB and Devesa SS (1994) Lung cancer. *Cancer* **75**: 191–202

US Department of Health and Human Services (1989) *Reducing the Health Consequences of Smoking. 25 Years of Progress.* A report of the surgeon general. U.S. Department of Health and Human Services, DHHS Publication No. (CDC) 89-8411

US Public Health Service (1964) *Smoking and Health*. Report of the advisory committee to the surgeon general of the public health service. US Department of Health, Education, and Welfare, PHS Publication No. 1103

Washimi O, Nagatake M, Osada H, Ueda R, Koshikawa T, Seki T, Takahashi T and Takahashi T (1995) In vivo occurrence of p16 (MTS1) and p15 (MTS2) alterations preferentially in non-small cell lung cancers. *Cancer Res* **55**: 514–517

Watanabe S, Tsugane S, Arimoto H, Shimosato Y, Suemasu K, Arai H and Urano Y (1987) Trend of lung cancers in the National Cancer Center of Japan and comparison with that of Japanese pathological autopsy records. *Jpn J Cancer Res* **78**: 460–466

Wynder EL and Graham EA (1950) Tobacco smoking as a possible etiologic factor in bronchiogenic carcinoma. *J Am Med Assoc* **143**: 329–336

© Cancer Research Campaign 1998